# Utilizing the reaction of degeneration test for individuals with focal paralysis

**DOI:** 10.1186/1749-7221-7-6

**Published:** 2012-07-25

**Authors:** Thomas J Holland

**Affiliations:** 1 Hunter College- Brookdale Campus, 425 E 25th St, Box 604, New York, NY, 10010-2590, USA

**Keywords:** Peripheral nerve lesion, Reaction of degeneration test, Electrodiagnosis

## Abstract

**Background and purpose:**

Neuromuscular electrical stimulation (NMES) is a modality sometimes used to help strengthen weak muscles. On occasion, however, the targeted muscles do not respond to the current delivered. No response to electrical stimulation should raise the consideration of unsuspected peripheral nerve damage. Two case studies are presented showing how absence of response was due to unsuspected peripheral neuropathy, which had not been considered in either of the original referral diagnoses.

**Case descriptions:**

The first individual sustained head trauma and did not respond to NMES to facilitate finger flexor contractions in the left hand. This prompted a reaction of degeneration test (R/D test) which revealed evidence of a median nerve lesion. The second individual presented with right ankle dorsiflexor and evertor paralysis following a right total hip replacement. The R/D test helped rule out a central nervous system lesion by revealing evidence of right peroneal nerve degeneration.

**Conclusion:**

The case reports show how clinical suspicion followed by simple R/D testing can be used to screen for nerve damage, prompting further electrodiagnostic work up of individuals with profound weakness and or paralysis.

## 

In 1850, the British physiologist Augustus V. Waller was the first to demonstrate that sectioning of a peripheral nerve results in a sequence of alterations resulting in degeneration of the distal portion of the severed axons [1]. Waller also showed that these changes occurred over time, requiring some days before degeneration became complete. These changes in the peripheral axons distal to the lesion became known as "Wallerian degeneration." Subsequently, Wilhem Erb [2], and others, showed that the contractile responses of muscles to different types of electrical stimulation depended upon whether the muscle was innervated or not. These studies also showed that the differences to the electrical stimuli between the innervated and denervated states also required a period of time after nerve section to occur. Since the time for Wallerian degeneration to become complete appeared to coincide with the time required for the electrical distinction between the innervated and denervated states to become apparent, the presence of the "abnormal" electrical responses became known as the Reaction of Degeneration, and the procedure to determine this reaction became known as Reaction of Degeneration (R/D) testing.

Peripheral nerve anatomical and molecular changes alter stimulation responses during the percutaneous application of short and long pulse duration currents over the lesioned nerve and denervated muscles [3-9]. Short duration pulsed currents (PC) with pulse durations between 50 microseconds and 1 millisecond will produce muscle contractions by depolarizing peripheral motor nerve. In contrast, if peripheral motor nerve is degenerated through trauma or disease, and time since nerve degeneration onset is greater than two weeks, denervated muscles require long duration PC (> 10 millisecond) for depolarization. Muscle fibers are not as electrically excitable as motor nerves and require a current with long duration. The inability to obtain direct muscle depolarization with short duration currents provides the basis for the R/D test and may provide the investigator an initial indication of nerve degeneration.

The R/D test has been used to assist in ruling out central nervous system (CNS) lesions as a source of paralysis [3,4]. Since the peripheral nerve remains intact with CNS lesions, the normal response to the short and long pulse duration currents will be preserved. Individuals with CNS induced paralysis use neuromuscular electrical stimulation (NMES) protocols with pulse durations between .2 to .4 milliseconds for such purposes as: maintaining range of motion, minimizing disuse muscle atrophy, improving motor control, orthotic substitution, and temporarily reducing spasticity [10]. In contrast, peripheral motor nerve denervations that require direct muscle activation require long duration currents [11]. Neuromuscular electrical stimulators commonly use electrical pulse widths that are <1 millisecond and therefore can not be used to stimulate peripherally denervated muscle. Short pulse duration NMES protocols for individuals with CNS lesions can not be used in cases in which muscle paralysis is also due to peripheral nerve degeneration.

The traditional R/D test was used prior to the establishment of electroneuromyography (ENMG) and nerve conduction velocity (NCV) studies to assist in the diagnosis of peripheral nerve lesions [6]. The R/D test is easy to perform and does not require any expensive and elaborate equipment. The test is relatively painless and quick and can be performed on individuals of all ages in both the clinical and home care setting. Electroneuromyographic and NCV studies are the first choice for a comprehensive electrodiagnostic evaluation of individuals with neuromuscular lesions. The R/D test is more of a qualitative test that should not replace quantitative electrodiagnostic procedures, but instead be used as a simple initial screening test for individuals with motor paralysis.

There is a lack of literature to assess the reliability and validity of R/D testing. The R/D test is qualitative in nature and requires the examiner to view if a contraction is present and to describe contraction quality (brisk versus sluggish). The need for long duration current stimulation for individuals with peripheral nerve injuries is reported in the research literature [12-16]. Required long duration current on denervated muscle is consistent with electrical stimulation responses following nerve degeneration as outlined in the R/D test. Even without formal R/D testing, diminished or absent responses to short duration PC indicates the possible need for further evaluation that includes ENMG / NCV studies.

The purpose of this article is to review the clinical application of R/D testing and present two case studies in which R/D testing served as a screening tool for individuals with CNS and PNS lesions. One of the following case reports demonstrates how R/D testing was used to detect a peripheral denervation not previously diagnosed in an individual with head trauma. In the other case study, R/D testing assisted in ruling out a CNS lesion and localizing the PNS lesion for an individual following total hip replacement surgery.

## Reaction of degeneration protocol

The traditional R/D test is performed by stimulating the lesioned nerve and two muscles normally innervated by that nerve [3]. A small probe electrode is placed on the suspected nerve and a large dispersive electrode either on the trunk or proximally on the extremity. Initially, short duration PC (< 1 millisecond) or high frequency (> 500 Hz) alternating current (AC) is delivered through the electrodes. Current intensity is slowly increased to elicit a tetanic muscle contraction as motor nerve threshold is reached (usually between 2–8 milliamps or a level comparable to the non-involved side). The same procedure is used on the motor points of proximal and distal muscles that normally receive their innervation from the lesioned nerve.

A response obtained with short duration current is reported as either no R/D or partial R/D. A strong tetanic contraction is graded as no R/D, while a weak or sluggish contraction when compared to the non-involved side implies an incomplete nerve degeneration and is classified as a partial R/D [3,6]. No response to short duration current is graded as either a full or absolute R/D and requires further testing with long duration current (> 10 milliseconds). The traditional R/D test uses a manually interrupted DC that provides a pulse duration greater then 100 milliseconds. When comparing innervated and denervated muscle contractions with long duration current, innervated muscle contraction is usually brisk while denervated muscle is more vermicular in nature. Response to long duration current after no response to short duration current is classified as a full R/D while no response with either current is graded as an absolute R/D. A full R/D indicates there is denervation but the muscle is intact and can be directly depolarized with long duration current stimulation. An absolute R/D is consistent with a severe denervation where there is muscle fiber atrophy and increased fibrotic tissue that cannot even respond to long duration current.

Before concluding R/D level, an important factor to consider is the polarity of the small stimulating electrode when using monophasic long duration PC or manually interrupted direct current (DC) waveforms. When using DC, the cathode (negative electrode) should be placed on the denervated muscle’s motor point when closing the circuit based on Pfluger’s law [6]. If no motor response occurs, the polarity should be switched (probe as anode and dispersive as cathode) and again the intensity slowly increased as the current is closed to attempt a long duration monophasic current induced contraction. A contraction with either anodal or cathodal stimulation would be graded as a full R/D. If no contraction occurs with long duration stimulation, the stimulator should be checked for proper functioning and a bipolar technique (two equal sized electrodes) may be applied to the area being investigated in order to concentrate the current while attempting a muscle contraction before making any R/D conclusion.

Some limitations exist for performing and interpreting the R/D test. Myopathic conditions in which the primary lesion is in the muscle (ie; muscular dystrophy, metabolic and endocrine myopathy) may result in weak or no contraction during R/D testing even when the peripheral nerve is intact. Additionally, severe edema may create electric impedance that limits direct nerve and muscle depolarization with transcutaneous stimulation. The R/D test is not valid if performed immediately following denervation due to the time course of nerve degeneration. At least two weeks may be required for the nerve to completely degenerate distal to the primary lesion site [6,8]. If the R/D test is performed too early and complete degeneration has not yet occurred, a false negative test may result. The R/D test should always be used in conjunction with patient history, current clinical presentation, and a comprehensive neurological examination including motor, sensory and reflex tests. Contemporary electrodiagnostic testing such as electroneuromyography (ENMG) and nerve conduction velocity (NCV) studies provide more detailed quantitative values to monitor neuromuscular integrity following an intial R/D screening.

## Case descriptions

### Patient A

The patient was a 37-year-old male who sustained closed head trauma after being struck by an automobile. He was initially hospitalized for medical stabilization that included surgical relief of increased intracranial pressure. Post-operatively the patient reacted specifically but inconsistently to stimuli. Two months following head trauma his cognitive functioning improved to an ability to display goal directed behavior with a dependence on verbal cues. The patient was transferred to a sub-acute rehabilitation facility to begin functional training and therapeutic exercise to improve motor control in the trunk and extremities.

### Examination

The patient was alert and oriented and able to follow instruction provided by the rehabilitation team. He required minimal assistance with bed mobility and stand pivot transfers. He ambulated approximately 200–300 feet without an assistive device but required close contact guard due to poor balance. He demonstrated a loss of active movement distally in both upper extremities (UE). The patient had minimal finger and wrist flexion and extension with increased flexor tone in the right UE. The left UE displayed no active wrist flexion, very minimal active wrist extension, no active finger movements and decreased muscle tone distally. He was unable to use the upper extremities for functional reaching and grasping activities due to motor control loss in the hands.

An increase in active finger flexion control was one goal established by the rehabilitation team. Neuromuscular electrical stimulation was incorporated in his treatment program to facilitate finger flexor contractions. A portable NMES unit (Empi Focus^a^) with a biphasic PC (pulse width of 300 microseconds) waveform was connected to electrodes placed on the finger flexor motor points. The stimulating frequency was set to 50 pulses per second, in an interrupted modulation (10 sec on: 30  off) with motor threshold stimulation. When attempting to produce NMES induced contractions, there was strong finger flexion on the right but no finger flexor contraction on the left. After attempting to alter stimulation parameters and electrode placement there was still no response in the left finger flexors. A formal R/D test was performed with an AC, DC, PC stimulator (Mettler model 207 A^b^). A large dispersive electrode was placed on the patient’s back and small stimulating probe on the flexor digitorum superficialis (FDS) motor point. The electrode placements and use of a dispersive pad avoided any electrical current concentration around the heart. Short duration stimulation was performed with a tetanizing (50 pulses/sec) symmetrical biphasic PC that was manually interrupted with the stimulating probe. The intensity was slowly raised to the expected motor threshold intensity (2 to 8 milliamps). After no response was noted the intensity was returned to zero and current output was switched to DC that was also manually interrupted with the stimulating probe. The small probe electrode placed on the FDS motor point was set as the cathode and the dispersive electrode as the anode. A weak contraction was noted as intensity reached 6 milliamps. The R/D test was performed on two other muscles innervated by the median nerve (flexor carpi radialis and abductor pollicis brevis) and similar results were obtained, no contraction with short duration current and weak contraction with DC. The patient was evaluated with an ENMG/NCV study that revealed a brachial plexus lesion that affected the left median nerve in the axilla. The rehabilitation team concluded that the patient’s brachial plexus injury may have been sustained during the initial trauma and further contributed to the loss of active left wrist and finger movements.

In this case, the R/D test allowed initial detection of a PNS lesion that was masked by the CNS induced paralysis. The patient was monitored during peripheral nerve regeneration and continued therapy with a home exercise program to maintain joint ROM, motor control, and muscle strength in the upper extremities. Detection of the PNS lesion assisted in formulation of the most time appropriate goals and treatment plans for the patient as peripheral nerve regeneration occurred.

### Patient B

The patient was a 71 year-old female referred for inpatient rehabilitation following a right total hip replacement (THR). Post-operatively she developed a deep vein thrombosis in the left posterior tibial vein for which she received anti-coagulation therapy. Her medical history was significant for mild hypertension, atrial fibrillation, hysterectomy following uterine cancer, and right THR due to osteoarthritis. Once she was cleared for rehabilitation, she presented with a lack of active right ankle dorsiflexion and given a standard plastic ankle foot orthosis (AFO) to prevent foot drag during ambulation. She began physical rehabilitation to increase mobility and strength in both lower extremities and achieve independence in activities of daily living, while maintaining total hip replacement precautions.

### Examination

The patient ambulated with a walker and a right posterior leaf spring orthosis with weight bearing as tolerated on the right lower extremity. She required close supervision on level surfaces and contact guard on uneven surfaces and elevations. The patient required minimal assistance with supine to short sitting transitions as part of bed mobility and performed stand pivot transfers with distant supervision. Table 1 provides a comparison of left and right ankle/foot muscle strength based on manual muscle testing. The patient demonstrated absent cutaneous sensation on the anterior surface of the right lower leg, dorsum of the foot, and between all the toes during sensory testing.

**Table 1:** Ankle Musculature Manual Muscle Testing Grades

**Muscle**	**Left Ankle**	**Right Ankle**
Tibialis anterior	0 / 5	4+ / 5
Gastrocnemius	4 / 5	4 / 5
Peroneus longus and brevis	0 / 5	4+ / 5
Tibialis posterior	4 / 5	4 / 5

A modified R/D test was performed on the tibialis anterior, peroneus longus and gastrocnemius muscles in both lower extremities three weeks following THR surgery. The test was performed with a manually interrupted symmetrical biphasic PC (pulse width 300 microseconds, frequency 50 pulses/sec, and motor threshold stimulation). A unipolar technique was used in which a large electrode was placed on the back and small stimulating probe on the investigated muscle’s motor point. The electrode placements and use of a dispersive pad avoided any electrical current concentration around the heart. All muscles responded with brisk contractions to short duration stimulation except the right tibialis anterior and peroneous longus in which there was no response. These two muscles were stimulated with a manually interrupted DC and a sluggish response was noted at an intensity of 5 milliamps for each. The R/D test results (Table 2), sensory testing, and motor testing were consistent with a deep peroneal nerve lesion. If a strong response with short duration current had occurred in all the lower leg muscles the paralysis would more likely be due to a CNS lesion since response to short pulse duration currents is contingent on an intact peripheral motor nerve.

**Table 2:** Reaction of Degeneration (R/D) Testing Results

**Muscle**	**Left Ankle**	**Right Ankle**
Tibialis anterior	Full R/D	No R/D
Gastrocnemius	No R/D	No R/D
Peroneus longus	Full R/D	No R/D

In addition to post-operative total hip management, the patient was referred to orthotic clinic for further orthotic evaluation. Gait training with her current orthosis was progressed to ambulation on all surfaces and elevations with both a walker and wide based quad cane. The patient was given a home exercise program that included closed kinematic and ROM / stretching exercises. The patient was eventually able to ambulate without the AFO as the previously denervated muscles became reinnervated following common peroneal nerve regeneration.

## Discussion

Upper and lower extremity muscle innervations can be initially evaluated with R/D testing. Knowledge of motor nerve innervations is required for the investigator to begin possible nerve degeneration localization. For example, short stimulus duration induced contraction in the thenar eminence muscles and only long duration induced contractions in the hypothenar eminence muscles is consistent with an intact median nerve and either a full or absolute ulnar nerve R/D. Proximal and distal muscles that share a common motor innervation can be stimulated to further localize denervation site. For example, normal short duration flexor carpi ulnaris induced contraction and no electrically induced abductor digiti minimi contraction indicates a possible ulnar nerve involvement somewhere in the forearm or wrist as opposed to upper arm or elbow.

Reaction of degeneration testing may assist in deciding if NMES is appropriate for an individual with motor paralysis. Short electrical pulse duration NMES is used to facilitate muscle contraction following CNS lesion [17-20]. The benefit of long duration stimulation of peripherally denervated muscle has been questioned. Some studies suggest excessive electrical stimulation to prevent muscle atrophy might actually inhibit the normal reinnervation process following a peripheral nerve lesion [11,21,22]. A quick R/D test may help the clinician to decide if electrical stimulation is indicated for an individual with motor paralysis.

Current waveforms indicated for muscle-strengthening protocols include biphasic PC, medium frequency AC, and high voltage monophasic PC [12-15]. An intact peripheral motor nerve is required when using these NMES waveforms, lack of a motor response during NMES (provided sufficient current intensity and appropriate electrode placement is used) may be an indication for further electrodiagnostic testing. As shown from the clinical cases presented, the R/D test can be used as a quick screening tool for initial detection of peripheral motor nerve denervation*.* Reaction of degeneration testing is not intended to replace well-established electrodiagnostic testing with ENMG. Instead, R/D testing may be a very simple, inexpensive, procedure that can be performed with any stimulator that has sufficient current intensity for motor stimulation. Small transcutaneous electrical stimulators (TENS) and NMES units or high voltage, and medium frequency stimulators can be used for short duration stimulation. Long duration current stimulators (low frequency AC, long duration PC, or DC) if available, could be used to differentiate between a full and absolute R/D in cases of complete nerve degeneration.

Further research is needed to assess R/D testing reliability between multiple investigators during different stages of nerve degeneration and reinnervation. In addition, criterion-related validity should be studied by performing the R/D test along with the more clinically practiced and accepted electrodiagnostic tests such as ENMG / NCV. The well established reactions to electrical stimulation following nerve degeneration, the availability of the required stimulators to most clinicians and the lack of expense and physical discomfort for the patient should stimulate future research into integrating the R/D test in clinical evaluation of individuals with motor paralysis.

## Conclusion

Differences in motor nerve and muscle responses to short and long electrical pulse duration current provide the basis for R/D testing. In conditions where the primary lesion involves peripheral nerve, the R/D test can be used as an adjunct in a comprehensive neurological evaluation. Delineation between CNS and PNS lesions and peripheral denervation localization is possible by stimulating multiple nerves and muscles with short or long duration current. Most clinics have stimulators that utilize these currents so they can be quickly applied as part of patient evaluation, if appropriate. Treatment goals and interventions can be more effectively formulated by having a better understanding of the neurological lesion location resulting in motor paralysis. Decisions pertaining to electrical stimulation use as a treatment modality can be made based on R/D testing results. The gold standard for electrodiagnostic testing continues to be ENMG and NCV studies and they can be used to help further establish the validity of R/D testing.

## Endnotes

^a^Empi Inc, Clear Lake Industrial Park, Clear Lake, SD 57226.

^b^Mettler Electronics, 1333 South Claudina St, Anaheim, CA 92805.

## Consent

Written informed consent was obtained from the two individuals who are presented as part of the case studies in this publication.

## Competing interests

The author declares that I do not have any competing interests.
